# Angiotensin IV does not exert prothrombotic effects *in vivo*^☆^

**DOI:** 10.1016/j.jmccpl.2025.100287

**Published:** 2025-02-12

**Authors:** Qifang Wu, Christine Gille, Florian Maderspacher, Bianca Hildebrand, Manuela Thienel, Sebastian Clauss

**Affiliations:** a Department of Medicine I, University Hospital, LMU Munich, Munich, Germany; b German Centre for Cardiovascular Research (DZHK), Partner Site Munich, Munich Heart Alliance (MHA), Munich, Germany; c Institute of Surgical Research at the Walter-Brendel-Centre of Experimental Medicine, University Hospital, LMU Munich, Munich, Germany; d Interfaculty Center for Endocrine and Cardiovascular Disease Network Modelling and Clinical Transfer (ICONLMU), LMU Munich, Munich, Germany

**Keywords:** Angiotensin IV, Thrombosis, Platelet aggregation, HGF, c-MET, PAI-1

## Abstract

Thrombosis and thromboembolism are serious clinical complications of cardiovascular diseases and are among the leading causes of mortality worldwide. Dysregulation of the renin-angiotensin system is associated with an increased incidence of thrombotic events. Angiotensin II (AngII) is known to enhance platelet aggregation, contributing to a prothrombotic state in patients. Important biological roles of other angiotensin peptides and their receptors have been shown, but their specific role in thrombus formation remains unclear. Recent evidence suggests a prothrombotic role of angiotensin IV (AngIV). To confirm the prothrombotic effects of AngIV and to further investigate AngIV-mediated mechanisms, we utilized osmotic minipumps to administer AngIV in mice continuously over 4 weeks. AngIV treatment did not induce thrombus formation in the heart, did not affect platelet numbers, and did not enhance platelet aggregation. HGF, c-MET, or PAI-1 expression levels in the heart were not affected by AngIV treatment in mice. Furthermore, we did not observe altered platelet aggregation of human platelets incubated with HGF. These findings indicate that AngIV does not regulate key prothrombotic mechanisms.

## Graphical abstract

**Figure:**
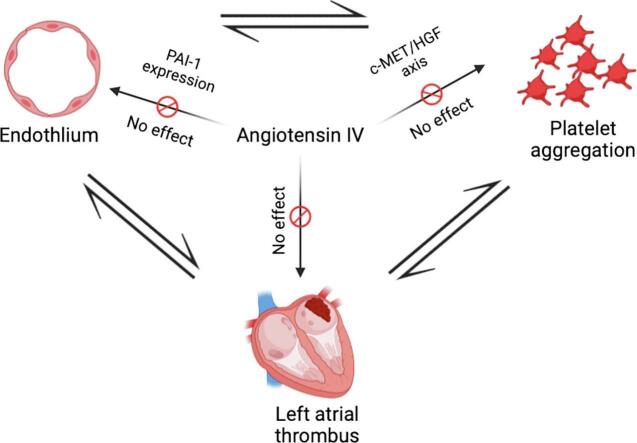

## Abbreviations

AngII: Angiotensin II
AngIV: Angiotensin IV
c-MET: Mesenchymal-epithelial transition factor
PAI-1: Plasminogen activator inhibitor-1
MI: Myocardial ischemia/infarction
AF: Atrial fibrillation
LAA: Left atrial appendage
RAAS: Renin-angiotensin-aldosterone-system
AngIII: Angiotensin III
IRAP: Insulin-regulated aminopeptidase
HGF: Hepatocyte growth factor
RT: Room temperature
PRP: Platelet rich plasma
PGI2: Prostaglandin
AUC: Area under the curve

## 1 Introduction

Thrombosis and thromboembolism represent leading causes of morbidity and mortality worldwide [1,2]. The pathogenesis of thrombus formation *in vivo* is primarily governed by Virchow's triad: hypercoagulability, venous stasis, and endothelial injury [3,4]. Heart disease and thrombosis/thromboembolism are closely intertwined, for example coronary artery thrombosis leading to myocardial ischemia/infarction (MI) [5,6], which is among the most common causes of atrial fibrillation (AF), which in turn predisposes to left atrial appendage (LAA) thrombus formation [3,7] with subsequent risk of embolization to the cerebral circulation resulting in ischemic stroke [8,9].

The increasing global burden of thrombotic events, alongside its complex and still incompletely understood pathogenesis and interplay with heart disease, highlights the critical need for further research into the mechanisms underlying thrombus formation in the heart. A deeper understanding of these pathways is essential for developing novel therapeutic strategies and improving clinical outcomes of heart disease.

Alterations of the renin-angiotensin-aldosterone system (RAAS) are involved in multiple cardiovascular diseases, *e.g.* arterial hypertension or thrombus formation [10,11]. Angiotensin II (AngII), the primary effector molecule of the RAAS, promotes a prothrombotic state and enhances platelet aggregation [12,13]. AngII undergoes enzymatic breakdown by aminopeptidases, resulting in the sequential formation of angiotensin III (AngIII) and subsequently Angiotensin IV (AngIV) [14]. Several studies suggest that AngIV may play an important role in thrombus formation by regulating multiple pathways. Ablation of the AngIV receptor (Insulin-regulated aminopeptidase, IRAP) has been demonstrated to reduce arterial thrombosis [15]. AngIV has also been demonstrated to stimulate the expression and secretion of plasminogen activator inhibitor-1 (PAI-1), a protein that stabilizes blood clots [16]. Furthermore, AngIV has been shown to inhibit the binding of the hepatocyte growth factor (HGF) to the mesenchymal-epithelial transition factor (c-MET), a tyrosine kinase receptor, which is highly expressed on platelets [17]. Since the binding of HGF to c-MET inhibits the GPIIb/IIIa-dependent platelet aggregation, AngIV may indirectly enhance platelet aggregation [17]. In sum, several studies suggest that AngIV may be a prothrombotic mediator, but a confirmation of this potential role especially within the heart is missing.

## 2 Methods

All measurements were performed in a blinded fashion.

### 2.1 Mouse model

All animal experimental procedures were approved and performed in accordance with the regulations of the Regierung von Oberbayern (ROB-55.2-1-54-2532-106-16), the EU Directive 2010/63/EU for animal experiments and the ARRIVE guidelines.

12-week-old C57BL/6 mice (Jackson Laboratories, Bar Harbor, ME, USA) were subjected to continuous subcutaneous infusion of AngIV or 0.9 % saline (NaCl) for 30 days using osmotic minipumps (Model #2004; Durect Corporation, Cupertino, CA, USA). The osmotic pumps were prepared to deliver AngIV (Phoenix Pharmaceuticals, Burlingame, CA, USA) at a dose of 0.96 μg/g/day or an equivalent volume of NaCl. On the day of implantation, mice were anesthetized with isoflurane (CP-Pharma, Burgdorf, DE) and positioned in the prone position on a surgical warming platform (Kent Scientific, CT, USA). Analgesia was achieved by Fentanyl (B. Braun, Melsungen, DE). An area of 2 × 4 cm in the back was shaved. A 1 cm dorsal incision was made to create a subcutaneous pocket, into which the filled pump was implanted. The incision was closed with 5-0 sutures (Ethicon Surgical Technology, Johnson & Johnson, NJ, USA) and disinfected with antiseptic solution. Mice were then placed under an infrared lamp for optimal recovery.

### 2.2 Murine platelet isolation

Murine blood was collected by cardiac puncture in anesthetized mice treated with AngIV or NaCl. Citrate-buffered blood samples were diluted 1:3 in Tyrode's buffer (pH = 6.5, 10 mM HEPES, 137 mM NaCl, 2.8 mM KCl, 12 mM NaHCO3, 5.5 mM glucose) and centrifuged at 70 × *g* for 20 min at room temperature (RT). Platelet-rich plasma (PRP) was further diluted with pH = 6.5 Tyrode's buffer and centrifuged at 600 × *g* for 10 min at RT. For further aggregation assays the platelet pellets were resuspended with Tyrode's buffer (pH = 7.4).

### 2.3 Human platelet isolation

Anticoagulated citrate blood was first centrifuged with 90 × *g* for 20 min at RT. Platelet rich plasma was then diluted 1:7 in modified Tyrode's buffer substituted with prostaglandin (PGI2, 500 ng/mL final concentration, Abcam, DE). PRP was then centrifuged at 450 × *g* for 10 min at RT. Pellets were re-suspended in Tyrode's buffer (pH = 7.4) for further analysis.

### 2.4 Light transmission aggregometry

Light transmission aggregometry was conducted using an Optical Aggregometer (Model 700, Chrono-Log Corporation, PA, USA) on washed platelets at a final concentration of 100,000 platelets/μL. The assay was performed in a total volume of 300 μL of Tyrode's buffer (pH = 7.4) containing 1 mM Mg^2+^ and Ca^2+^. Platelet aggregation was triggered using the following agonists: for murine samples and human samples, 10 μg/mL fibrillar collagen (Chrono-Log Corporation, PA, USA) and 0.02 U/mL or 0.1 U/mL thrombin (Chrono-Log Corporation, PA, USA) for human and murine platelet aggregation were tested, respectively. When specified, samples were preincubated with HGF (ImmunoTools, Friesoythe, DE) at various concentrations and incubation times. Aggregation responses were quantified by measuring the area under the curve (AUC) and maximum aggregation.

### 2.5 Western blot

Isolated, washed human platelets or murine dissected cardiac tissue were lysed in RIPA buffer (Sigma-Aldrich, USA) containing protease inhibitors (Protease Inhibitor Cocktail, Sigma-Aldrich, USA) and phosphatase inhibitors (PhosSTOP, Roche, Switzerland). Protein concentration was quantified using the BCA assay (Thermo Fisher Scientific, MA, USA), and equal amounts of protein were resolved *via* SDS-PAGE under reducing conditions, followed by transfer to nitrocellulose membranes using a wet transfer system (Bio-Rad Laboratories, CA, USA). Membranes were blocked with 5 % BSA (Sigma-Aldrich, MO, USA) in TBS-T and incubated overnight at 4 °C with a rabbit monoclonal anti-PAI-1 antibody (1:1000, Cat# ab66705, Abcam, Cambridge, UK), anti-HGF polyclonal antibody (1:1000, Cat#PA5-115354 Thermo Fisher, MA, USA), anti-c-Met antibody (1:1000, Cat#ab51067, Abcam, Cambridge, UK), phosphor c-Met (1:1000, Cat#3077, Cell Signaling Technology, MA, USA). After washing, HRP-conjugated secondary antibodies (Cell Signaling Technology, MA, USA) were applied for 1 h, and chemiluminescent signals were developed using ECL reagents (Merck, Darmstadt, Germany) and captured with a ChemiDoc MP Imaging System (Bio-Rad Laboratories, CA, USA).

To confirm equal protein loading, membranes were stripped and reprobed with anti-GAPDH antibody (1:5000, ab190304, Abcam, Camb, UK). Band intensities were quantified using ImageJ software (NIH, USA), and data were normalized to GAPDH signals to ensure comparability.

### 2.6 Statistical analysis

Data were analyzed using GraphPad Prism 8 software (GraphPad Software, San Diego, CA). Normality of data was assessed with the D'Agostino-Pearson omnibus test. Where appropriate, statistical comparisons were made using either an unpaired student's *t*-test or One-way ANOVA.

## 3 Results

### 3.1 Angiotensin IV does not promote platelet aggregation in vivo

**Fig. 1:**
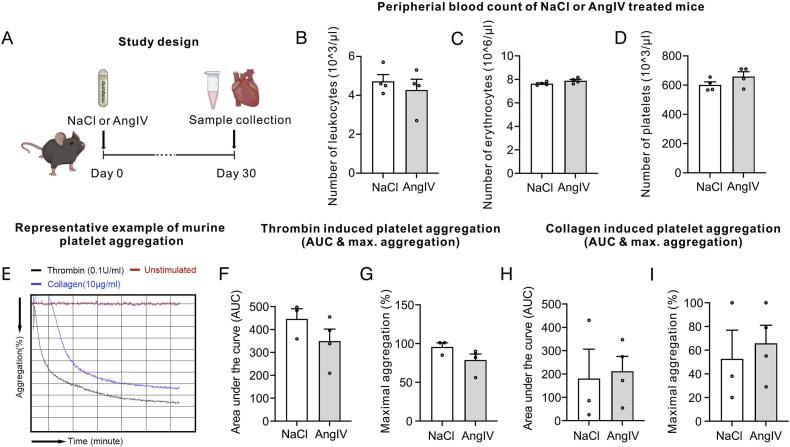
(A) Study design. C57BL/6 mice were treated for 30 days with NaCl or AngIV administered by osmotic minipumps. Murine leukocyte (B), erythrocyte (C) and platelet (D) numbers were obtained following 30 days of NaCl or AngIV treatment (*n* = 4 mice per group, circles indicate individual mice). (E-I) Platelet aggregation in NaCl (*n* = 3) and AngIV-treated (n = 4) mice in response to different stimuli (thrombin at 0.1 U/mL (F,G) or collagen at 10 mg/mL (H,I)) was assessed by analyzing the area under the curve (AUC, F,H) and maximal aggregation (%, G,I), circles indicate individual mice. (E) Representative example of murine platelet aggregation. Data shown as MEAN ± SEM, *P*-values were determined using unpaired Student's *t*-tests. *p < 0.05, **p < 0.01.

To investigate whether AngIV promotes thrombus formation in the heart, we employed a mouse model with continuous AngIV administration *via* osmotic pumps for 30 days (Fig. 1A). AngIV administration did not induce (macroscopically) visible thrombus formation in the murine heart, nor did it alter the numbers of leukocytes (Fig. 1B), erythrocytes (Fig. 1C), or platelets (Fig. 1D). To assess whether AngIV influences platelet activity, we performed platelet aggregation assays using light transmission aggregometry (Fig. 1E-I). Platelets from AngIV-treated mice showed no significant differences in aggregation compared to control mice when stimulated with agonists such as 0.02 U/mL or 0.1 U/mL thrombin (Fig. 1E-G) or 10 μg/mL collagen and (Fig. 1E,H-I).

### 3.2 HGF does not affect platelet aggregation and AngIV does not influence expression of HGF/c-MET

**Fig. 2:**
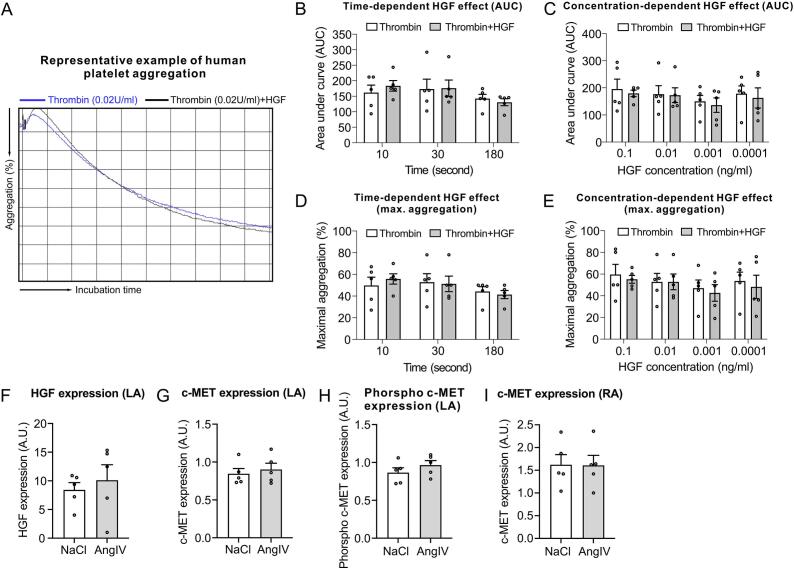
(A-E) Effects of HGF on human platelet aggregation were assessed by analyzing the area under the curve (AUC, B,C) and maximal aggregation (%, D,E) in the presence of various concentrations of HGF (C,E) and incubation times (B,D), *n* = 5 healthy donors, circles indicate individual donors. (A) Representative example of human platelet aggregation. Protein expression of HGF (F), c-MET (G,I) and phospho-c-MET (H) in the atria of mice treated with NaCl or AngIV (n = 5 mice per group, circles indicate individual mice). Data are shown as MEAN ± SEM, *P*-values were determined using unpaired Student's *t*-test. *p < 0.05, **p < 0.01.

To investigate whether AngIV plays an indirect prothrombotic role *via* the HGF/c-MET signaling pathway, we evaluated the anti-thrombotic effects of HGF on human platelet aggregation under various HGF concentrations and incubation times (Fig. 2A-E). While previous studies have suggested that HGF transiently inhibits platelet activity after 10, 30, or 180 s of incubation, respectively, we did not observe a similar inhibitory effect in our experiments under the same conditions.

Given the clinical relevance of atrial thrombus formation as a frequent complication in cardiovascular disease, we further examined whether AngIV affects expression of HGF or c-MET and whether it regulated c-MET phosphorylation in atria of mice after 30 days of AngIV administration. As shown in Fig. 2F-I, no significant differences in HGF, c-MET or phosphorylated c-MET expression were observed.

### 3.3 PAI-1 expression does not display site-specific variation within the heart

**Fig. 3:**
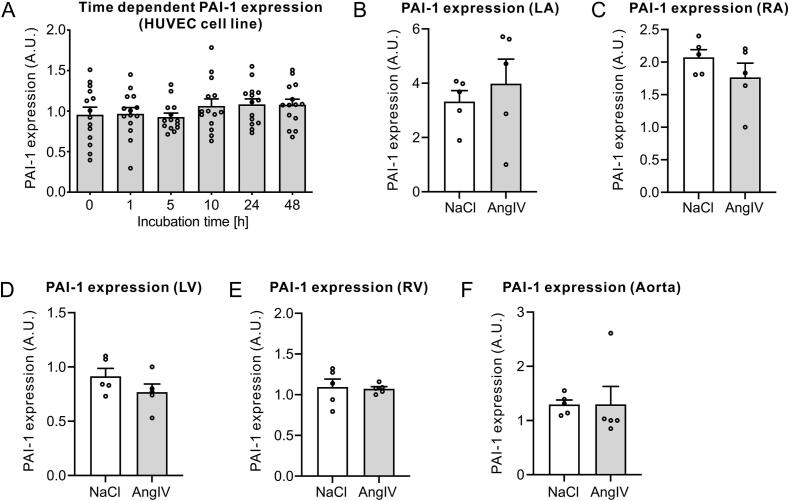
(A) Protein expression of PAI-1 in HUVEC cells treated with AngIV at different incubation durations (*n* = 14 replicate wells per time point, circles indicate individual wells). Data are shown as MEAN ± SEM, *p < 0.05, **p < 0.01, *P* values were determined using One-way ANOVA. (B–F) Protein expression of PAI-1 in various cardiac regions of mice treated with NaCl or AngIV (n = 5 mice per group, circles indicate mean values of 3 technical replicates per individual mice). Data are shown as MEAN ± SEM, *P*-values were determined using unpaired Student's *t*-test. *p < 0.05, **p < 0.01.

To evaluate a potential prothrombotic effect of AngIV by regulating PAI-1 expression on endothelial cells, we treated HUVEC cells with AngIV. PAI-1 expression was not affected by AngIV treatment over time (Fig. 3A). Since a recent study showed an organ-specific PAI-1 expression in the endothelium, we elucidated whether AngIV regulates expression of PAI-1 in different regions of the heart (Fig. 3B-F). Mice treated with AngIV for 30 days did not show altered PAI-1 expression in atria (Fig. 3B-C), ventricles (Fig. 3D-E) or in the aorta (Fig. 3F).

## 4 Discussion

Thrombus formation is a major contributor to the morbidity and mortality associated with heart disease, arising from a complex pathogenesis involving hypercoagulability, endothelial dysfunction, and platelet activation. AngII, the central regulator of the renin-angiotensin-aldosterone-system (RAAS), promotes clot formation by enhancing the expression of procoagulant factors, such as tissue factor and plasminogen activator inhibitor-1 (PAI-1). Additionally, AngII induces endothelial damage through oxidative stress and activates platelets by binding to AT1 receptors on their surface. RAAS-targeted therapies have been shown to effectively reduce thrombosis in both patients and rat models [18], highlighting the critical role of RAAS in thrombotic processes.

There is a growing body of evidence that AngII is not the only mediator of RAAS effects since it is enzymatically cleaved into multiple angiotensin peptides which have been demonstrated to have specific functions [[19], [20], [21], [22], [23]]. One of these peptides is angiotensin IV (AngIV) which gained some interest as a potential mediator of heart disease as several studies have demonstrated that AngIV activates nuclear transcription factor-kappaB (NF-κB) and related proinflammatory genes in vascular smooth muscle cells, contributing to vascular inflammation, atherosclerosis, and the pathogenesis of cardiovascular diseases [24]. AngIV also stimulates atrial natriuretic peptide (ANP) secretion in a dose-dependent manner, serving as a compensatory mechanism in heart failure [25]. Additionally, it improves myocardial dysfunction in ischemia-reperfusion (I/R) injury [26,27] and modulates cardiac function by affecting LV systolic performance, enhancing pressure sensitivity to volume changes, and accelerating myocardial relaxation. These findings indicate that AngIV is involved in multiple mechanisms/signaling cascades in the context of various cardiovascular diseases. Thus, AngIV may regulate prothrombotic mechanisms under pathologic conditions which we cannot exclude based on our findings obtained in healthy mice. Furthermore, some studies suggest AngIV as a prothrombotic mediator since it has been shown to enhance expression of PAI-1 both in endothelial cells from animal models and in human endothelial cells [28,29], and ablation of its receptor IRAP results in reduced arterial thrombosis [15]. Given that another study suggests that AngIV analogs inhibit the interaction between HGF and its receptor c-MET [30,31], a potential indirect prothrombotic effect seems possible since binding of HGF to c-MET prevents platelet aggregation [32]. Thus, the goal of our study was to confirm AngIV as a prothrombotic mediator in the heart and to further investigate underlying AngIV-regulated mechanisms.

Since AngIV is a derivative of AngII, we used a dose of AngIV that corresponds to doses of AngII used in prior studies [33], thus avoiding excessive doses which might result in different effects due to potential binding to alternative receptors as shown for other angiotensin peptides before [34]. Our findings revealed that continuous AngIV administration for 30 days did not induce thrombus formation in the murine heart, did not affect platelet numbers, platelet activity, or expression of HGF, c-MET and PAI-1, though effects on vascular alterations in diabetic mice have been shown after 30 days of AngIV treatment [34]. Although our results suggest that AngIV does not mediate prothrombotic effects under physiologic conditions, prothrombotic effects in the context of cardiovascular diseases cannot be excluded. Furthermore, we could not confirm the inhibitory effects of HGF on human platelet aggregation and could not observe any effect of AngIV on PAI-1 expression on endothelial cells.

A previous study reported that human platelet aggregation can be transiently inhibited by HGF [32]. However, we could not confirm this observation using the same experimental conditions. The only difference was the platelet preparation method which may partly explain the discrepant findings. While we used washed platelets to minimize contamination with plasma proteins such as HGF [35], the original study employed gel-filtered platelets, which are known to retain an aggregation phenotype more comparable to platelet-rich plasma (PRP) [36]. This highlights an important methodological consideration for analyzing platelet function and may explain discrepant findings between our study and the previous report. Additionally, HGF and c-Met expression levels in the left atrium were similar in AngIV-treated mice compared to saline-treated mice. In sum, our data suggest that AngIV does not regulate the HGF/c-Met signaling pathway on an expressional or on a functional level.

AngIV has been demonstrated to stimulate the release of plasminogen activator inhibitor-1 (PAI-1) in coronary artery endothelial cells *in vitro* [29], potentially stabilizing thrombi by inhibiting fibrinolysis. We assessed the potential effect of AngIV on PAI-1 expression using HUVEC cells but did not observe any effect on PAI-1 expression following AngIV treatment. Additionally, analysis of murine heart tissue samples showed no region-specific upregulation of PAI-1 after 30 days of AngIV treatment, indicating that AngIV does not regulate PAI-1.

## 5 Limitations

To assess the potential thrombotic effects of AngIV, we administered a 30-day treatment at a functional dose in healthy mice under steady-state conditions, ensuring that any observed effects could be attributed solely to the treatment with AngIV (rather than to any disease-related condition). While no thrombotic activity was detected under these conditions, this approach was essential to determine whether AngIV alone contributes to thrombosis independently of pathological stimuli. However, we also acknowledge several limitations in our study.

Firstly, although we selected the dose based on established pharmacological principles to ensure functional relevance while avoiding extreme doses that could introduce confounding variables, alternative doses - particularly higher or lower concentrations - could yield different effects, potentially through interactions with additional receptor pathways as reported for other angiotensin peptides before [34]. Future studies exploring a broader dose range would provide a more comprehensive understanding of AngIV's thrombotic potential. Secondly, the 30-day treatment duration was determined for its established efficacy in assessing vascular alterations as reported before [33]. While a shorter exposure should have revealed at least some thrombotic manifestations if AngIV had a strong prothrombotic effect, we cannot rule out the possibility that prolonged treatment could yield different outcomes. Practical constraints, including minipump capacity and ethical considerations, limited our ability to test longer durations; however, future studies incorporating extended treatment periods could provide further clarity. Finally, our study was conducted in healthy mice, allowing us to assess AngIV's direct effects without the confounding influence of an underlying disease. Given that thrombotic risk is often exacerbated in pathological conditions, it remains essential to also investigate AngIV's effects in disease models where platelet function and coagulation pathways may be altered. Future studies in cardiovascular disease models will be critical for translating these findings into a clinically relevant context.

Despite these limitations, our study provides valuable insights by demonstrating that AngIV does not independently trigger thrombosis under physiological conditions. However, given the complex and still incompletely known biological roles of AngIV, further research is warranted to fully elucidate its effects under varying experimental conditions, including different doses, treatment durations, and pathological states.

## 6 Conclusion

Overall, our findings challenge the proposed prothrombotic role of AngIV and its involvement in platelet activation/aggregation, HGF/c-MET signaling, and PAI-1 regulation. Despite evidence linking AngIV to thrombosis in previous studies, we demonstrate that AngIV does not promote thrombus formation, platelet numbers or aggregation, or regional changes of the expression of prothrombotic mediators in the heart. Furthermore, we could not confirm previous study results suggesting that HGF may inhibit platelet aggregation. Although our study results indicate no role of AngIV in regulating intracardiac thrombosis formation, further studies are warranted to further elucidate AngIV-regulated mechanisms.

## CRediT authorship contribution statement

**Qifang Wu:** Writing – review & editing, Writing – original draft, Investigation, Formal analysis. **Christine Gille:** Writing – review & editing, Investigation, Formal analysis. **Florian Maderspacher:** Investigation, Formal analysis. **Bianca Hildebrand:** Methodology. **Manuela Thienel:** Methodology. **Sebastian Clauss:** Writing – review & editing, Supervision, Resources, Project administration, Funding acquisition, Conceptualization.

## Funding

This study was supported by the LMU Förderprogramm für Forschung und Lehre (FöFoLe, to F.M.).

## Declaration of competing interest

The authors declare that they have no known competing financial interests or personal relationships that could have appeared to influence the work reported in this paper.

## Appendix A Supplementary data

Supplementary material Image 1
