# Supplementary material for: Angiotensin IV does not exert prothrombotic effects in vivo
Source: J Mol Cell Cardiol Plus. 2025 Feb 12;11:100287. doi: 10.1016/j.jmccpl.2025.100287 (PMC11871494; doi:10.1016/j.jmccpl.2025.100287)
Supplement: Supplementary file 1 — Supplementary material [file mmc1.docx]

**Angiotensin IV does not exert prothrombotic effects *in vivo***

**Qifang Wu ^a,b,c,d *^, Christine Gille ^a,b,c *^, Florian Maderspacher ^a,b,c^, Bianca Hildebrand^a,d^, Manuela Thienel ^a,b,d^ , Sebastian Clauss ^a,b,c,d^**

**Supporting information**

**Western blot results related to figure2F:
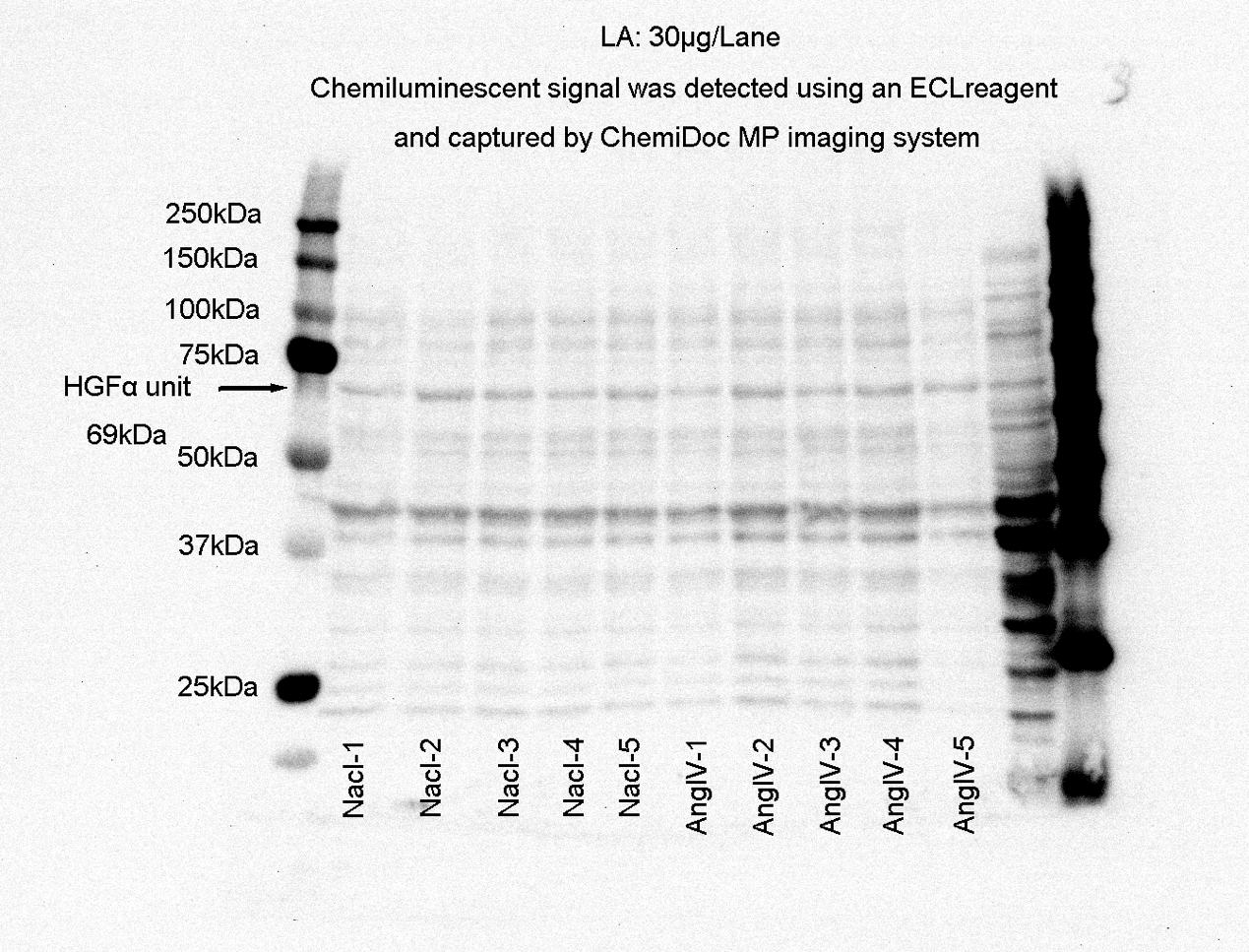
**

**
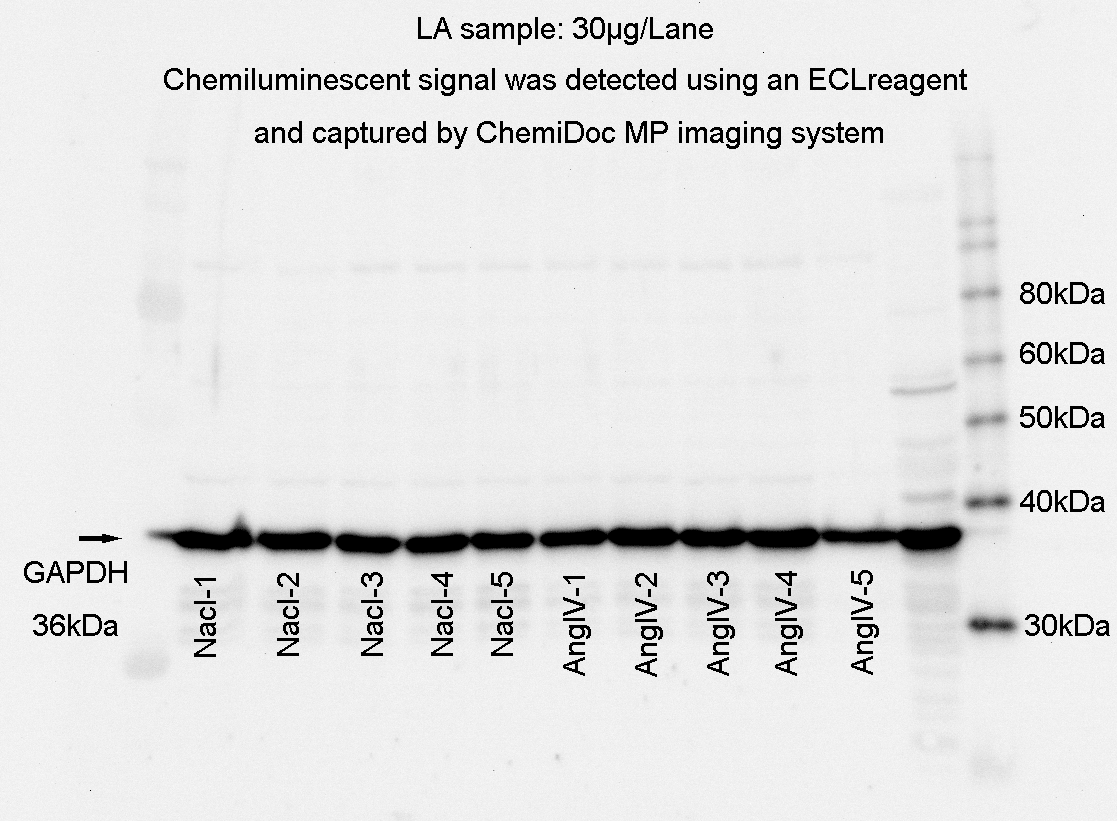
**

**Western blot results related to figure2G:**

**
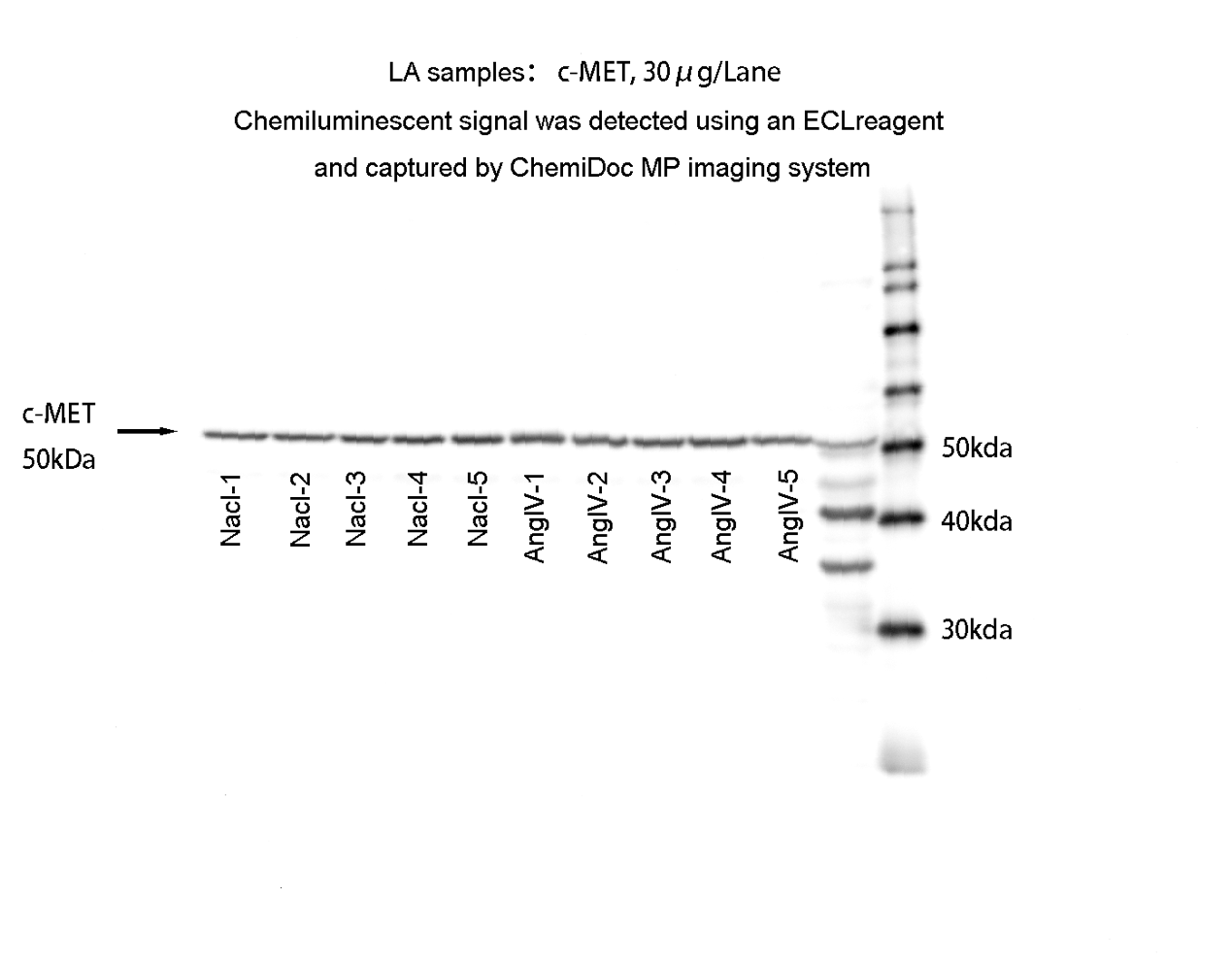

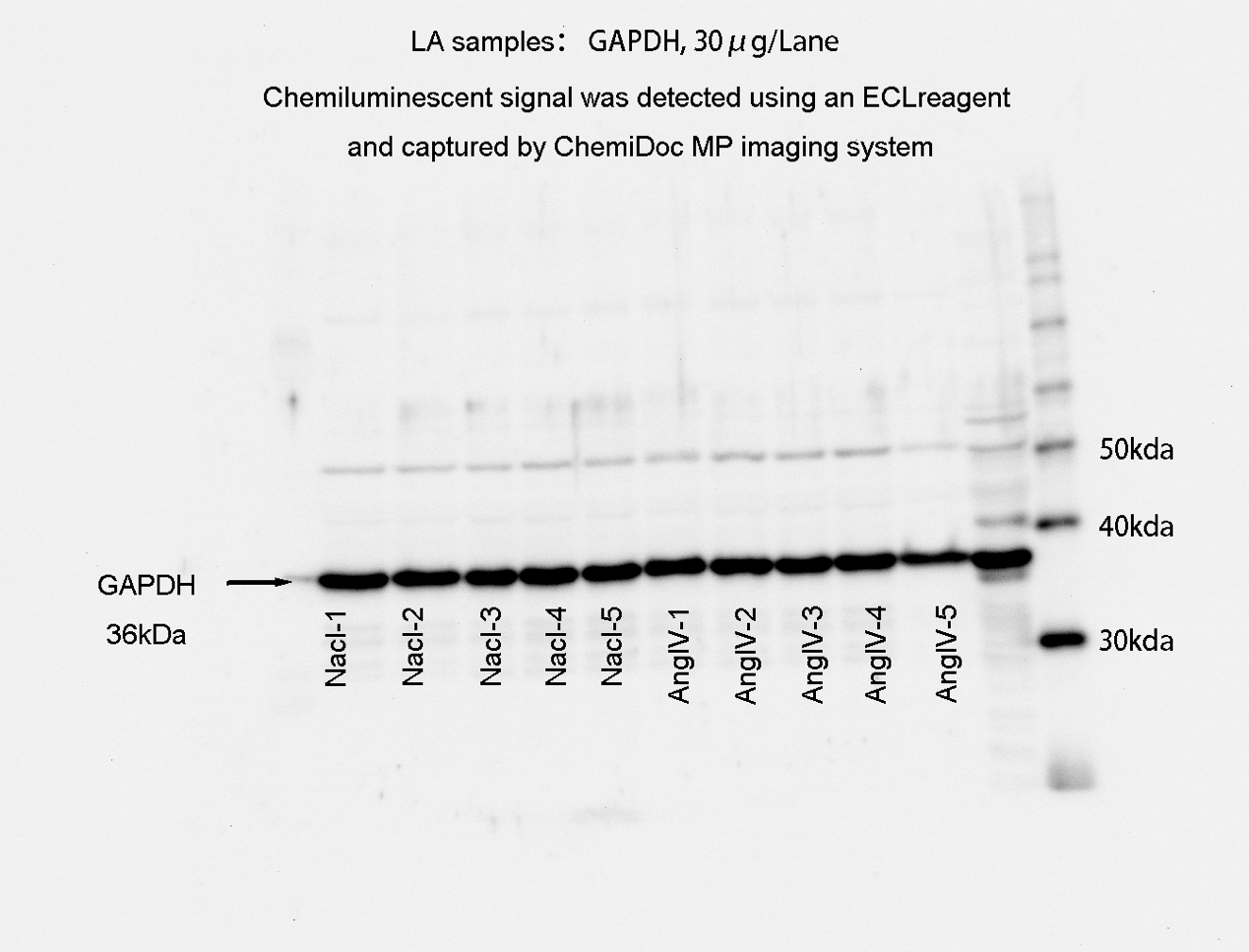
**

**Western blot results related to figure2H:**

**
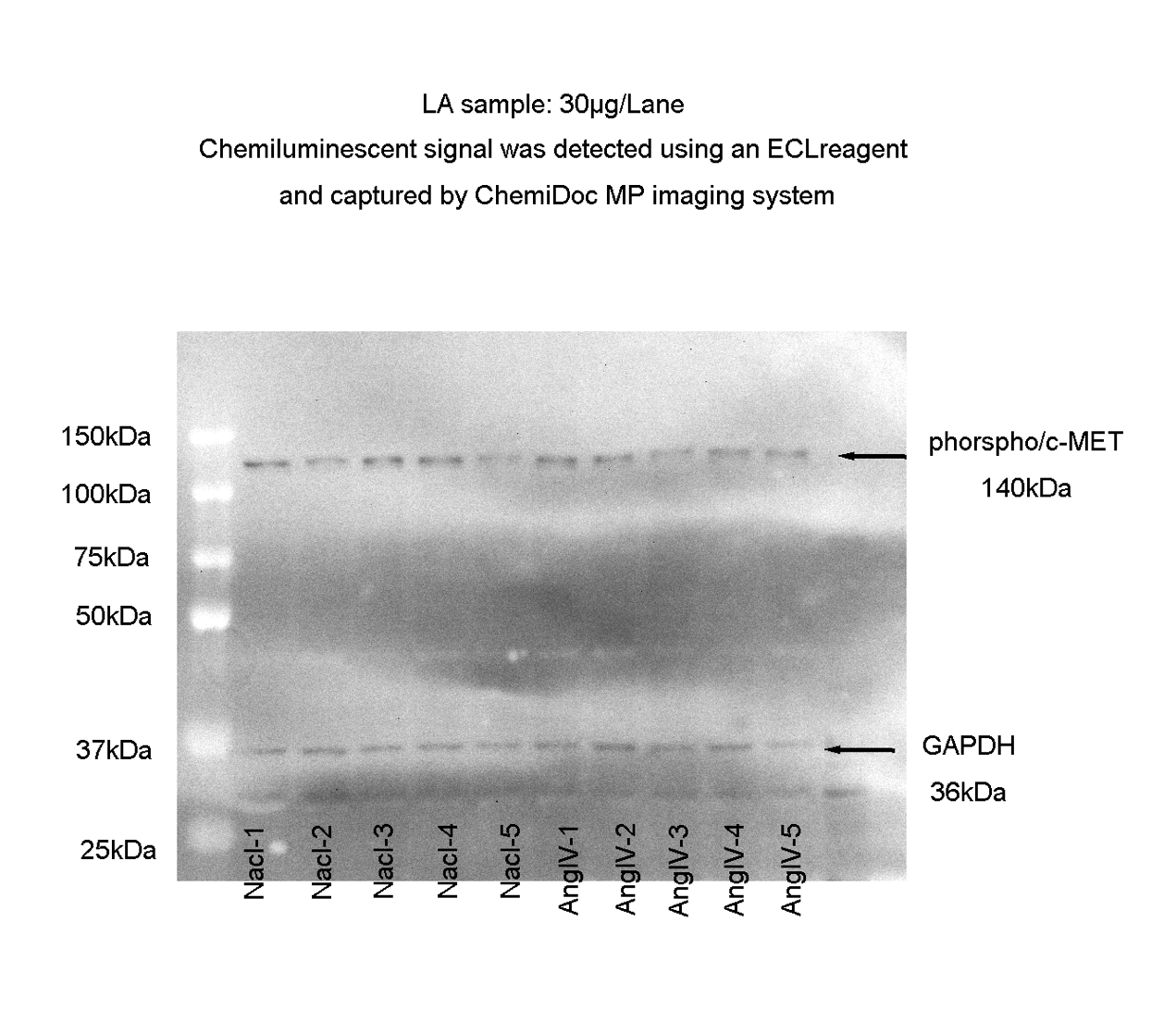
**

**Western blot results related to figure2I:**

**
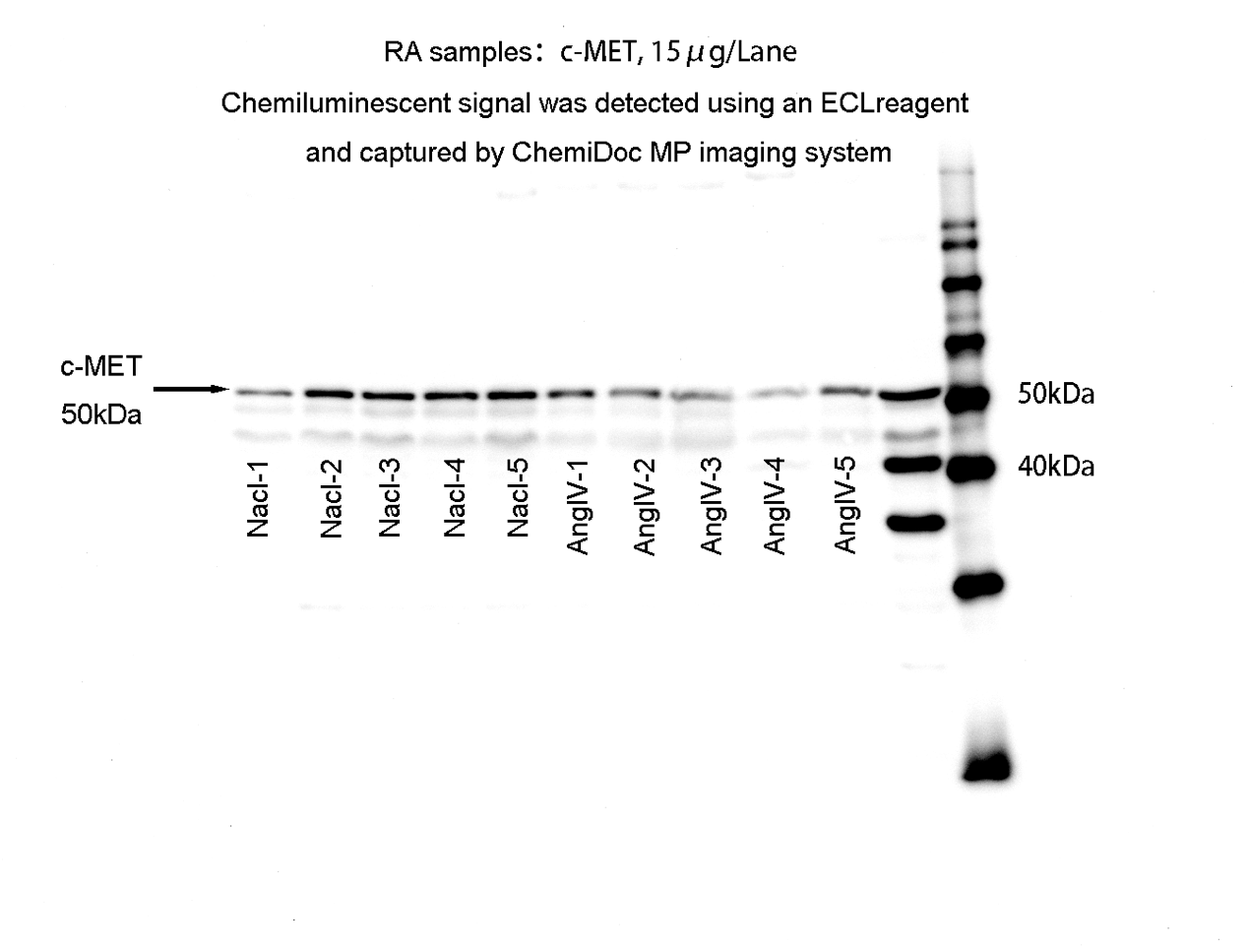

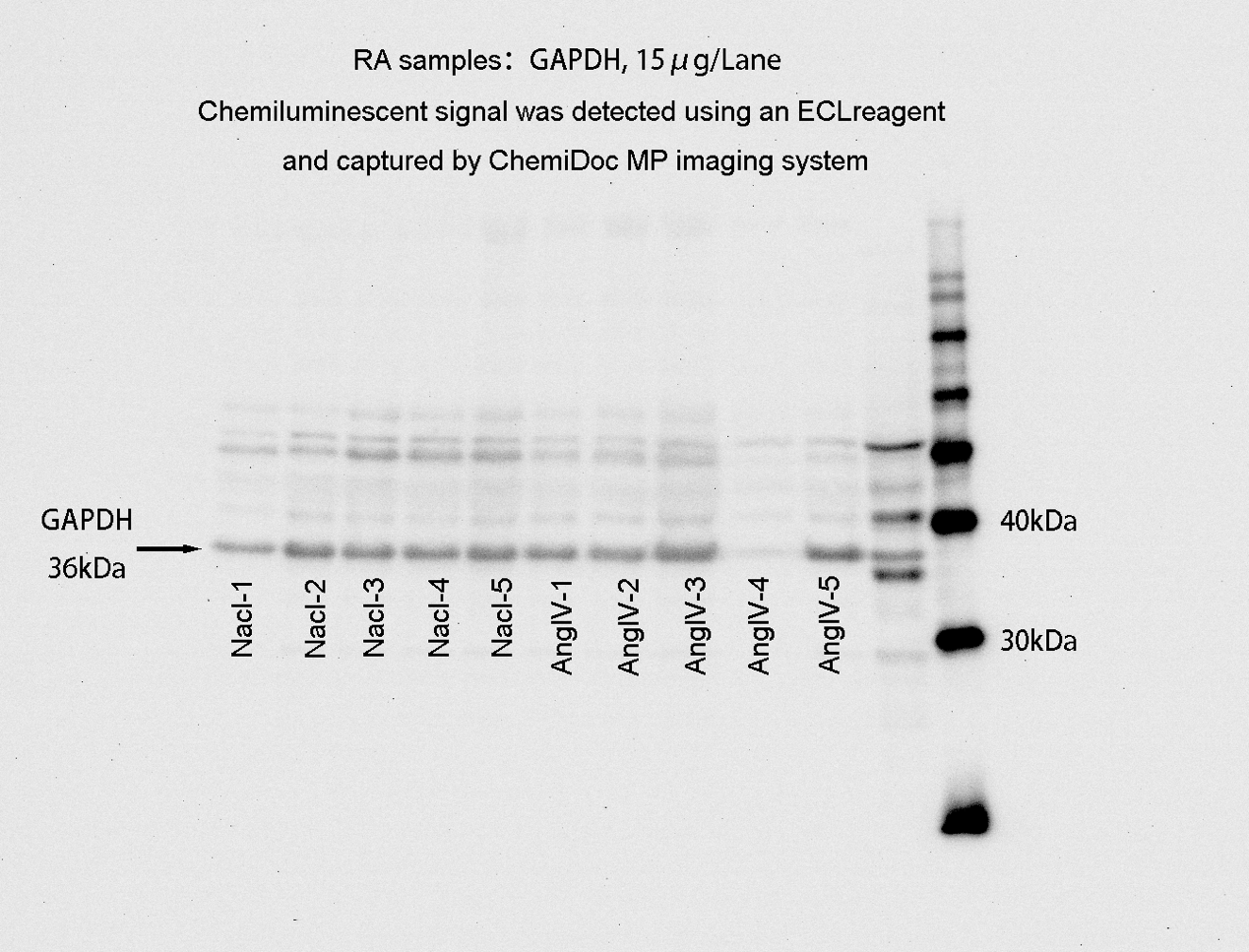
**

**Western blot results related to figure3A:**

**
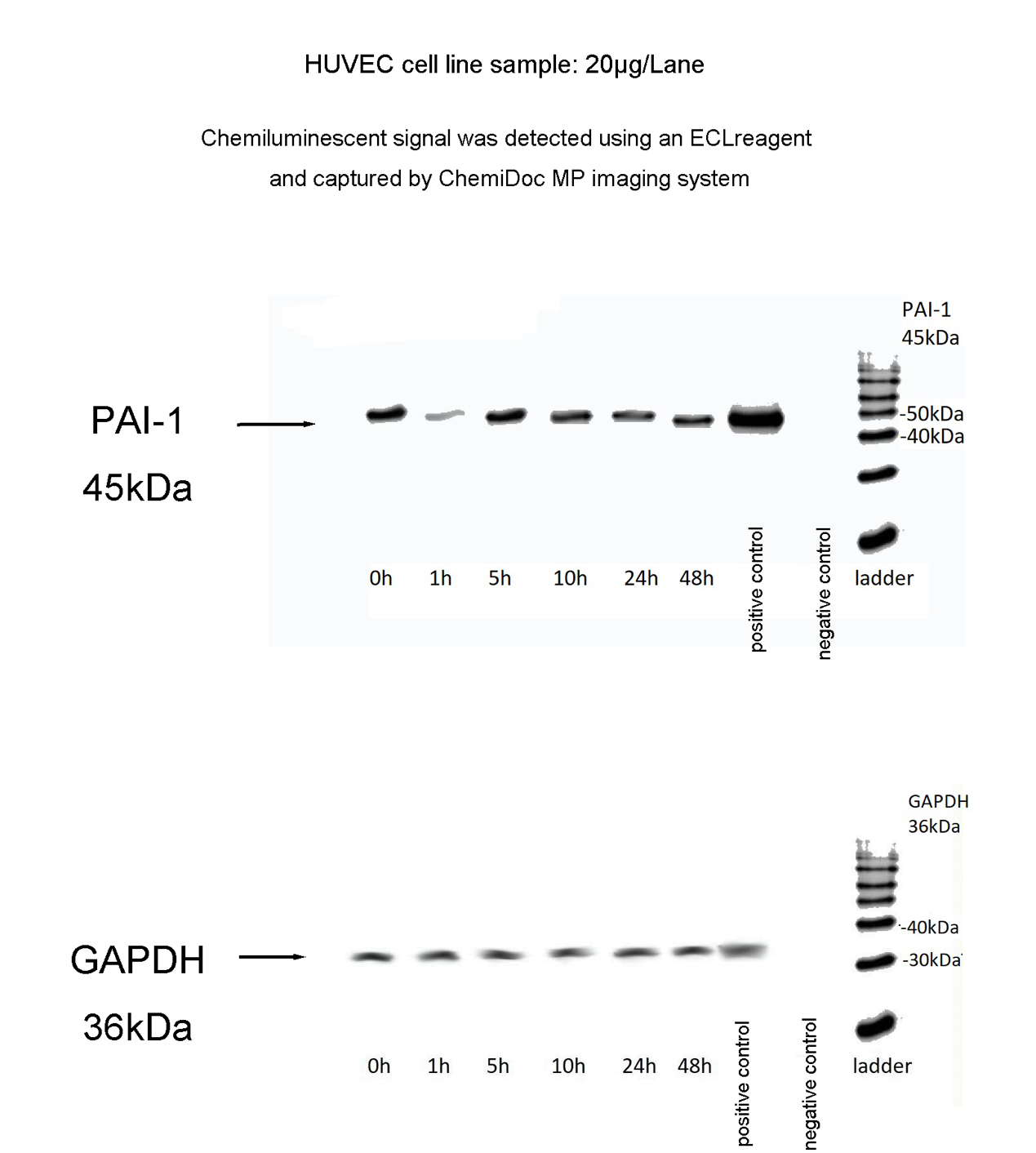

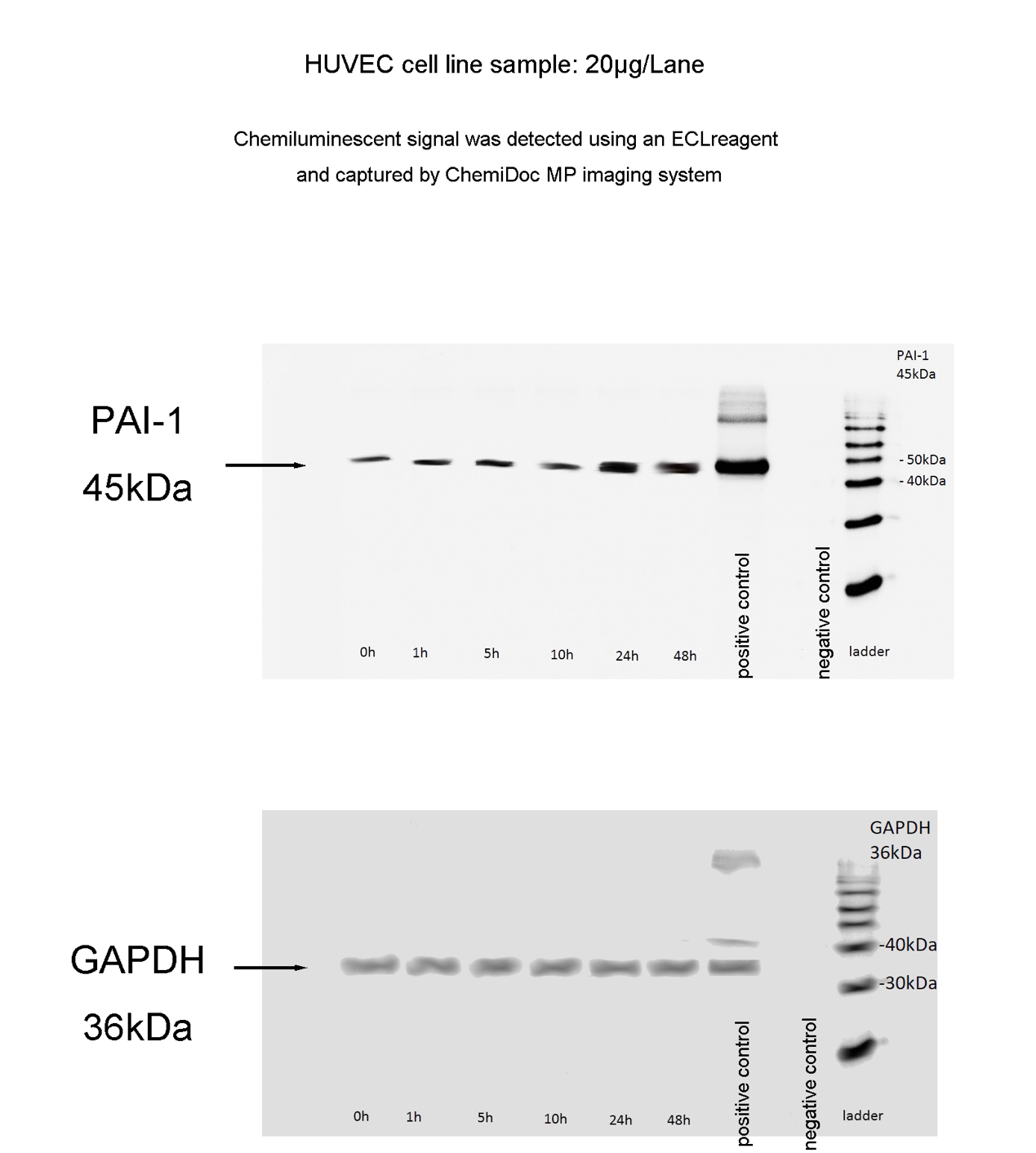

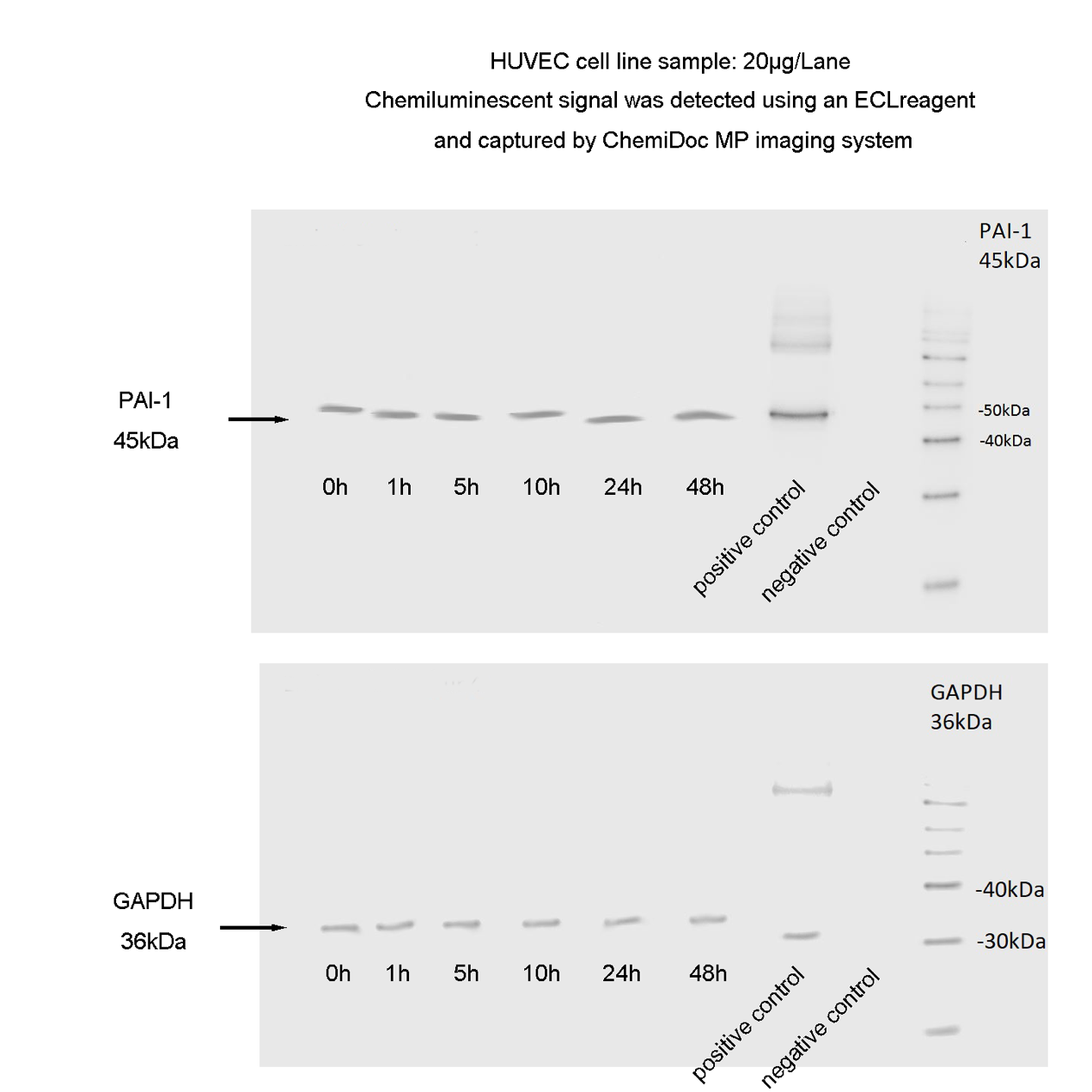

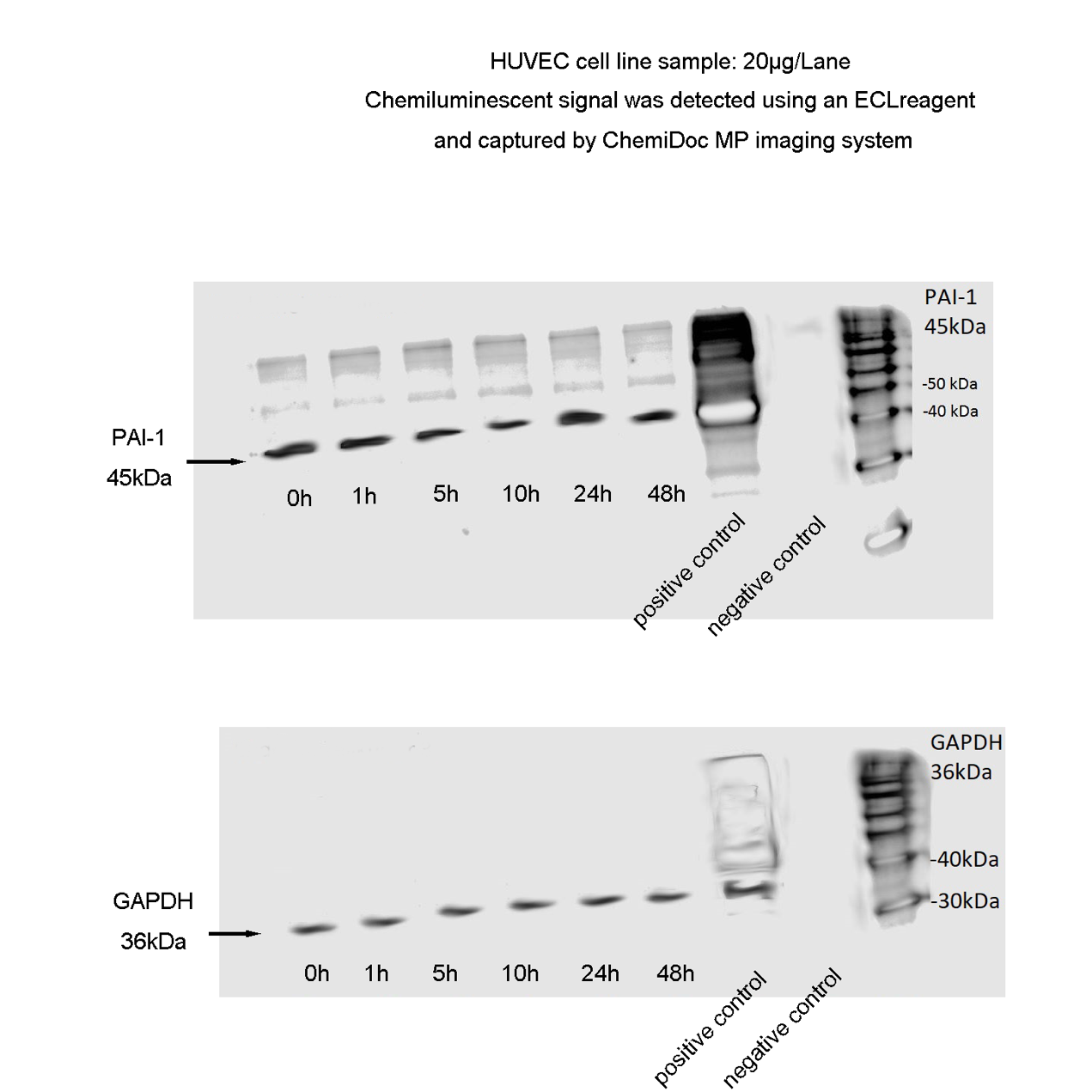

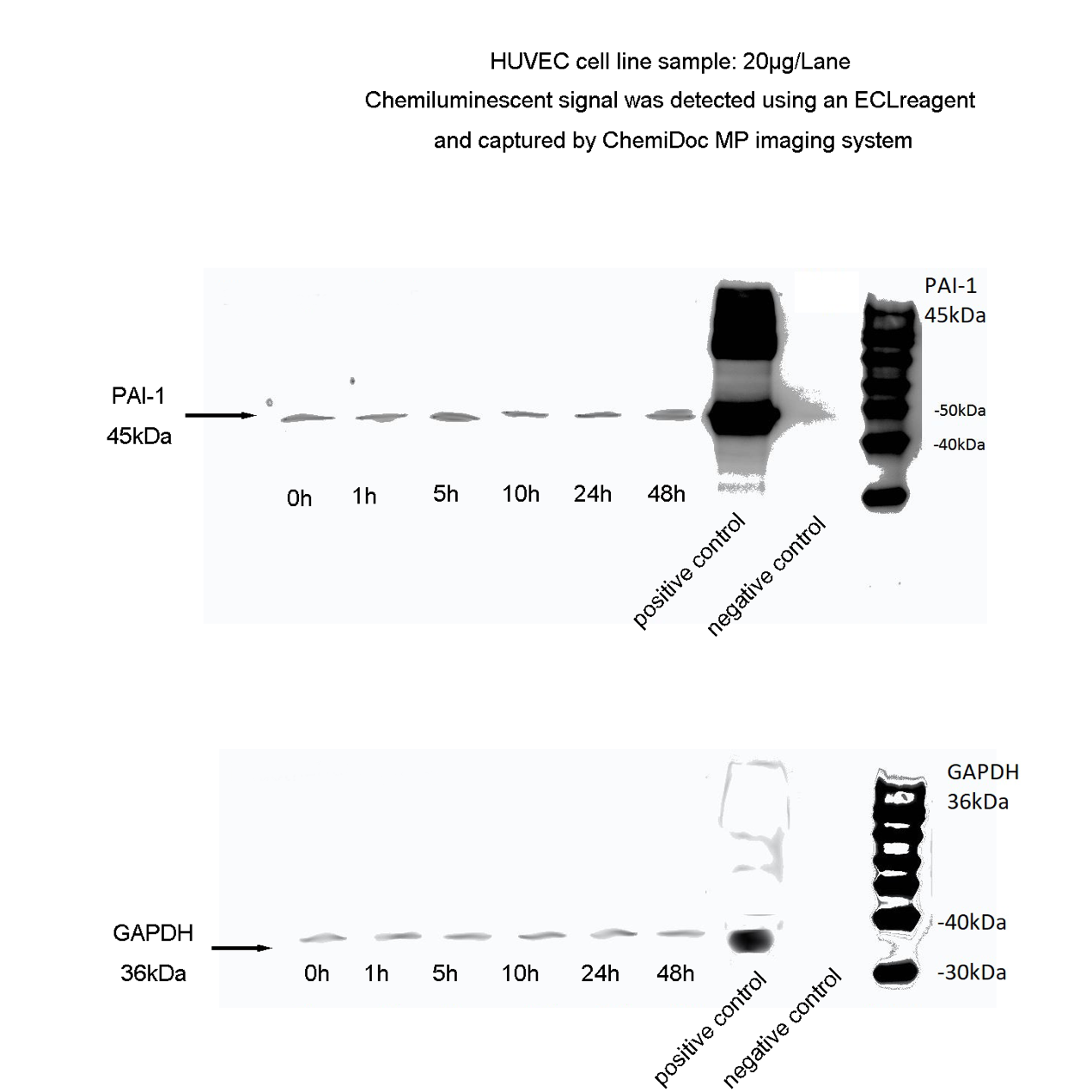

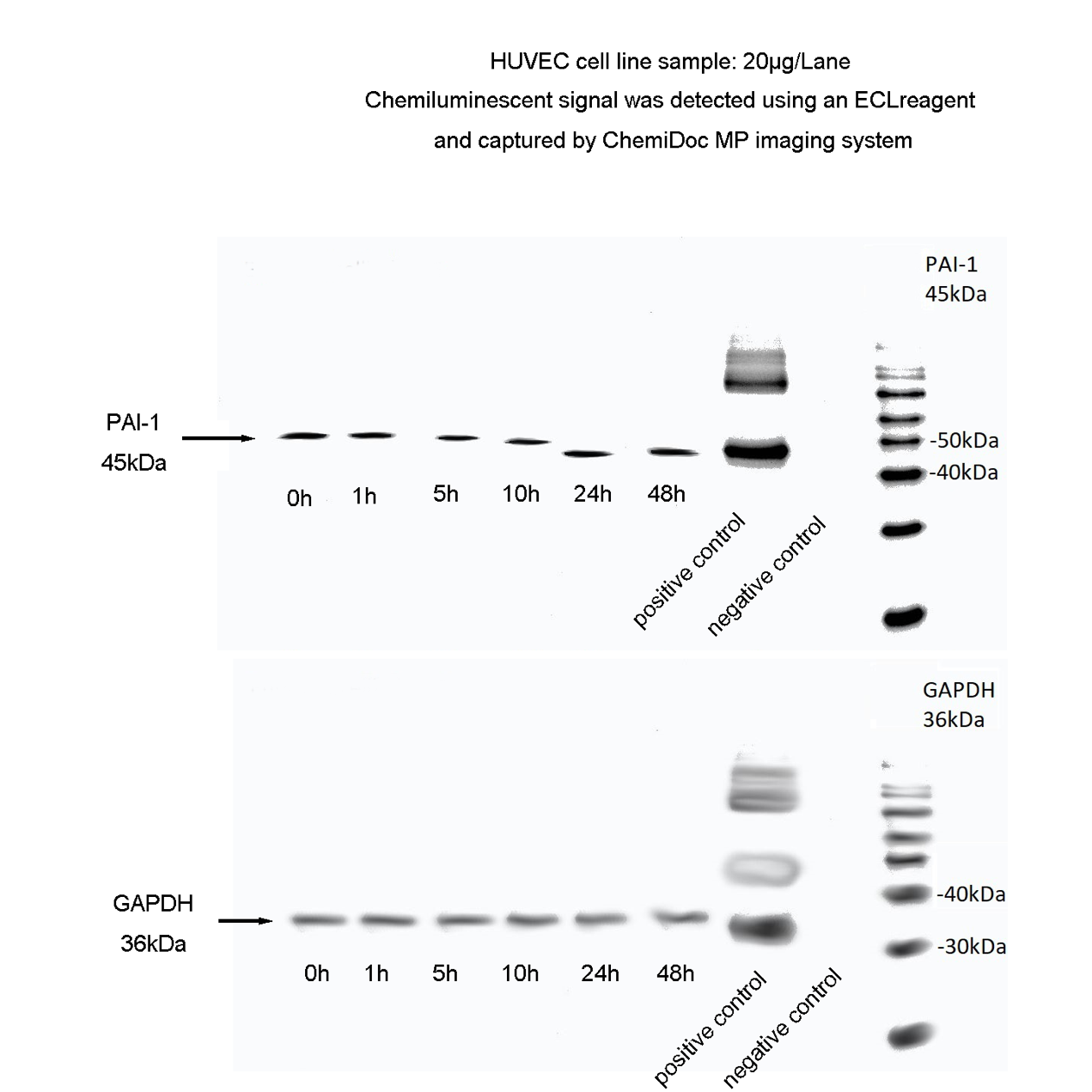

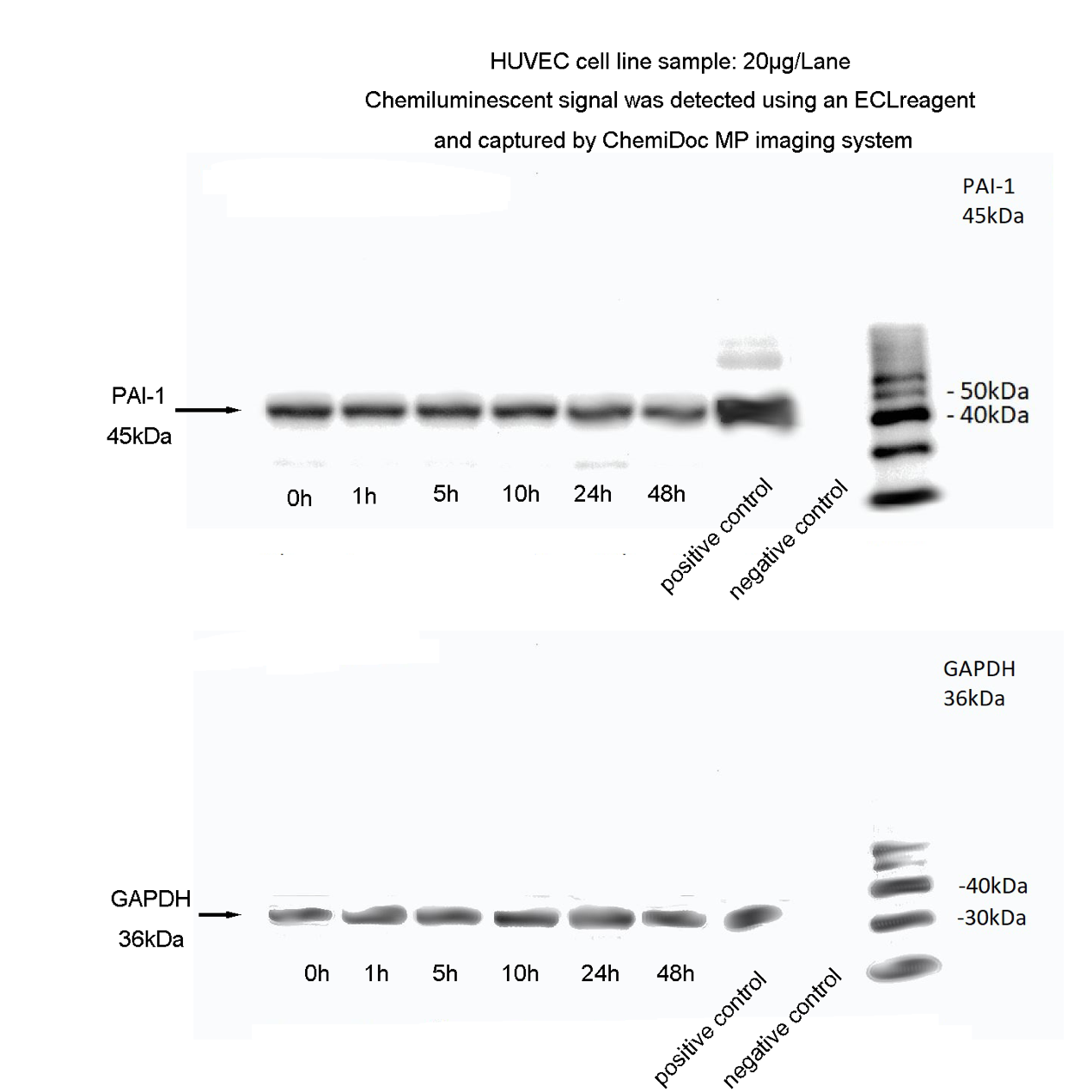

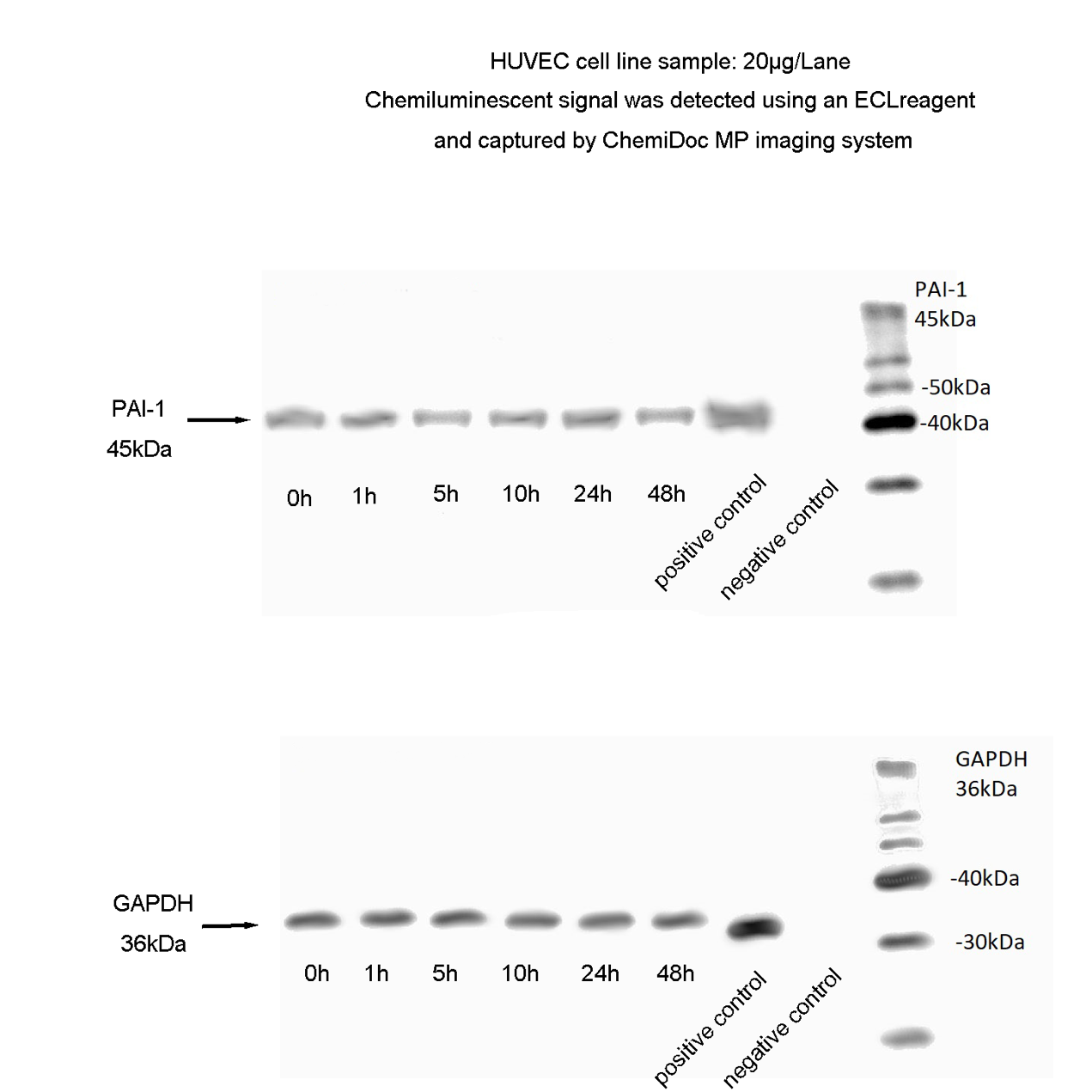

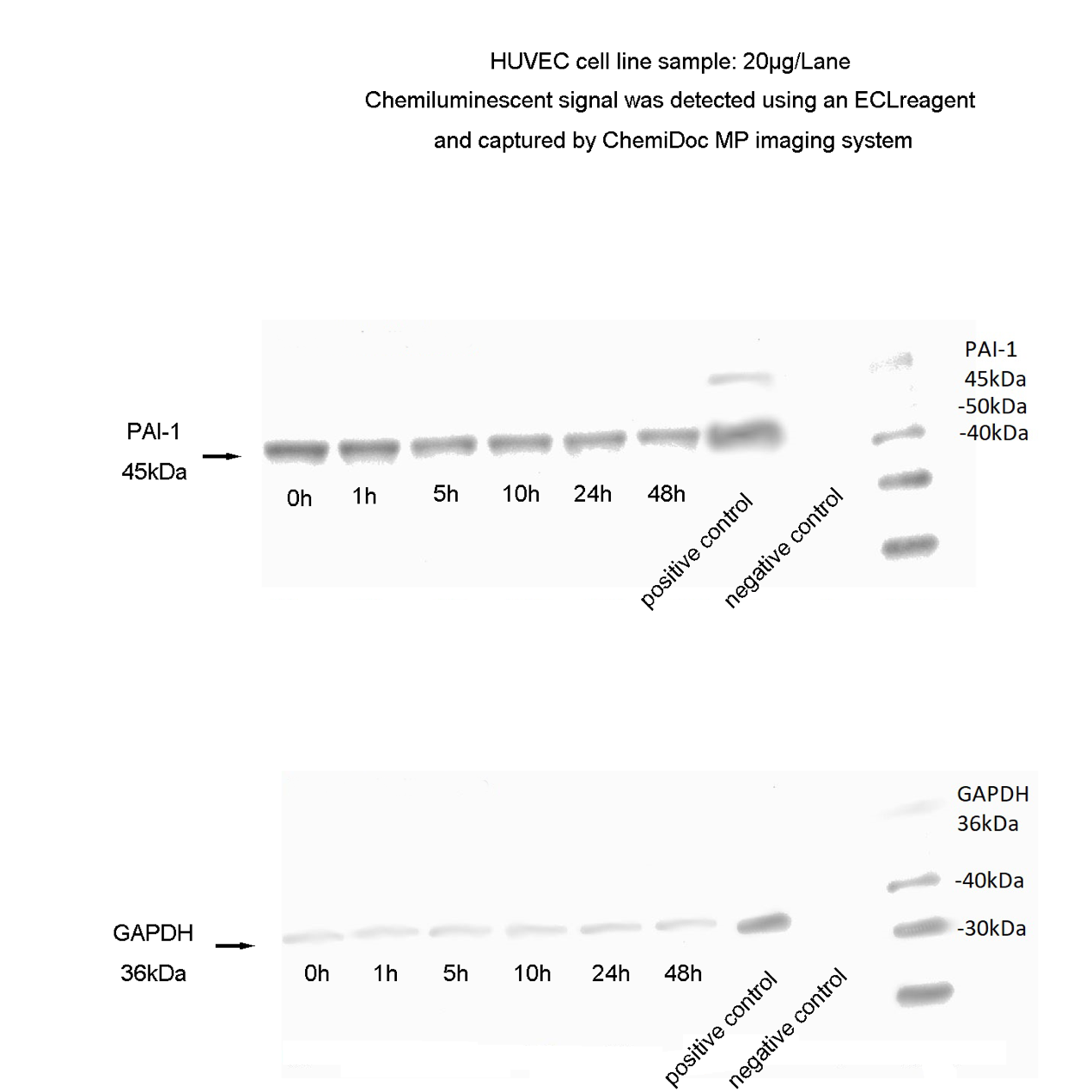

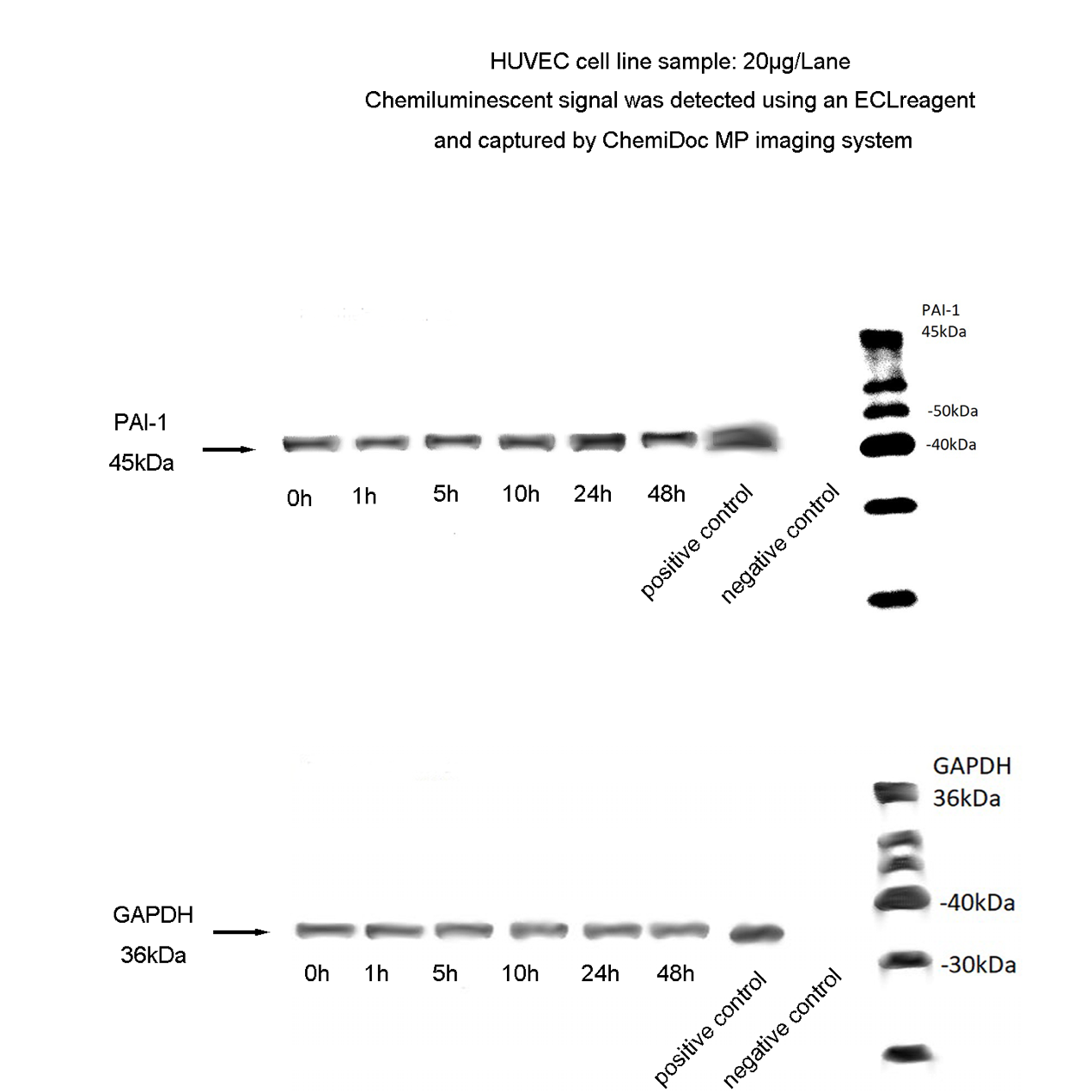
**

**Western blot results related to figure3B:**

**
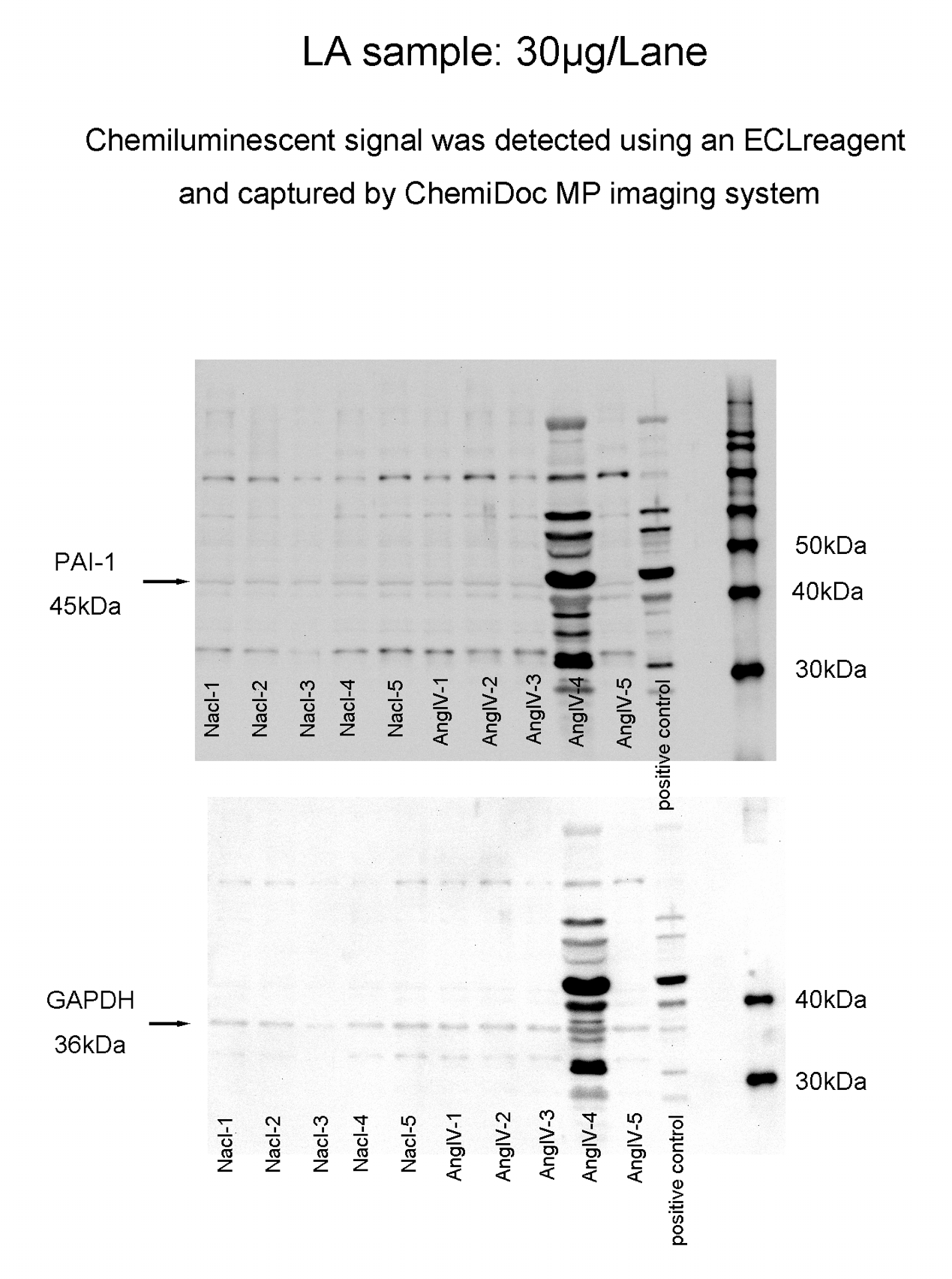
**

**Western blot results related to figure3C:**

**
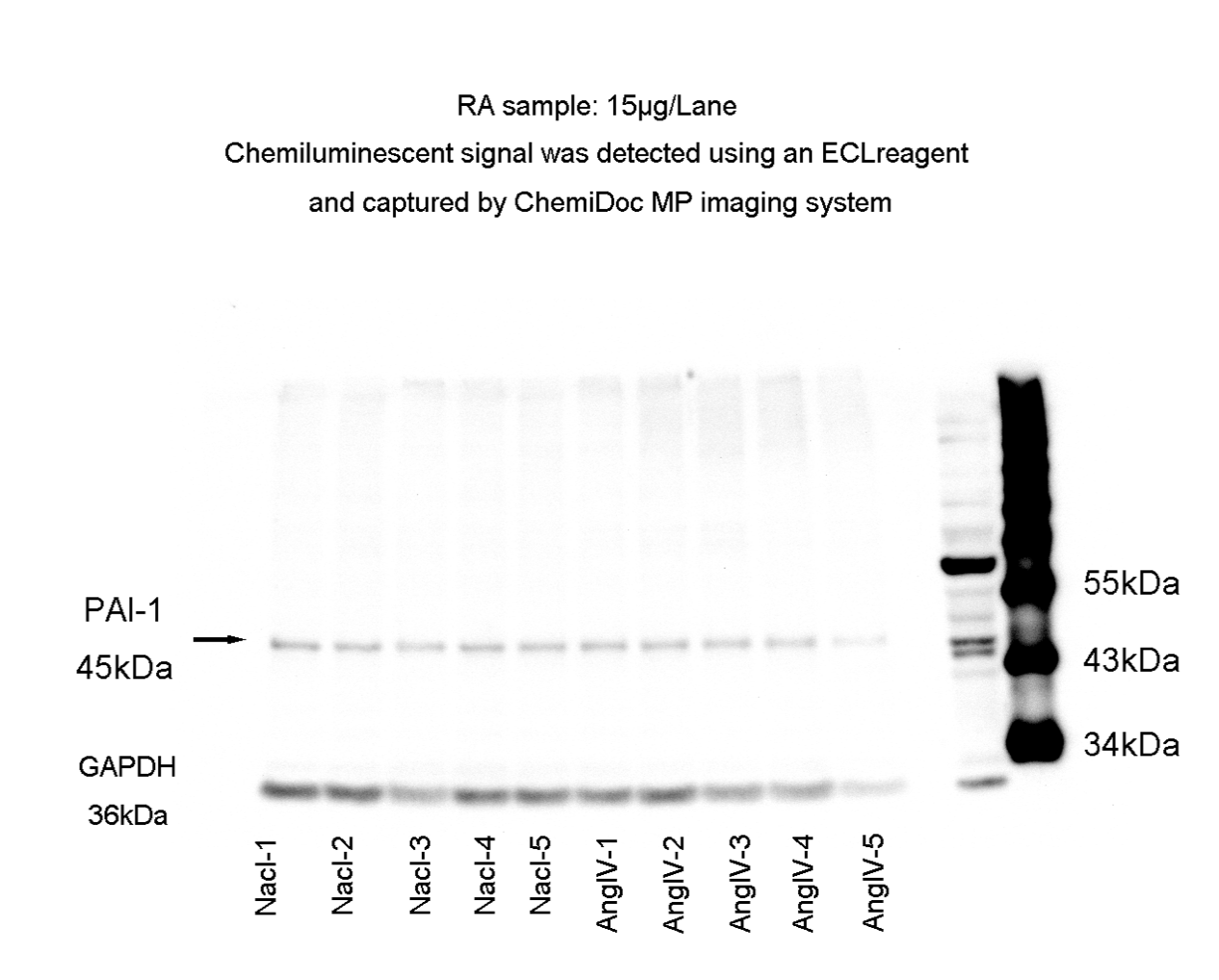
**

**Western blot results related to figure3D:**

**
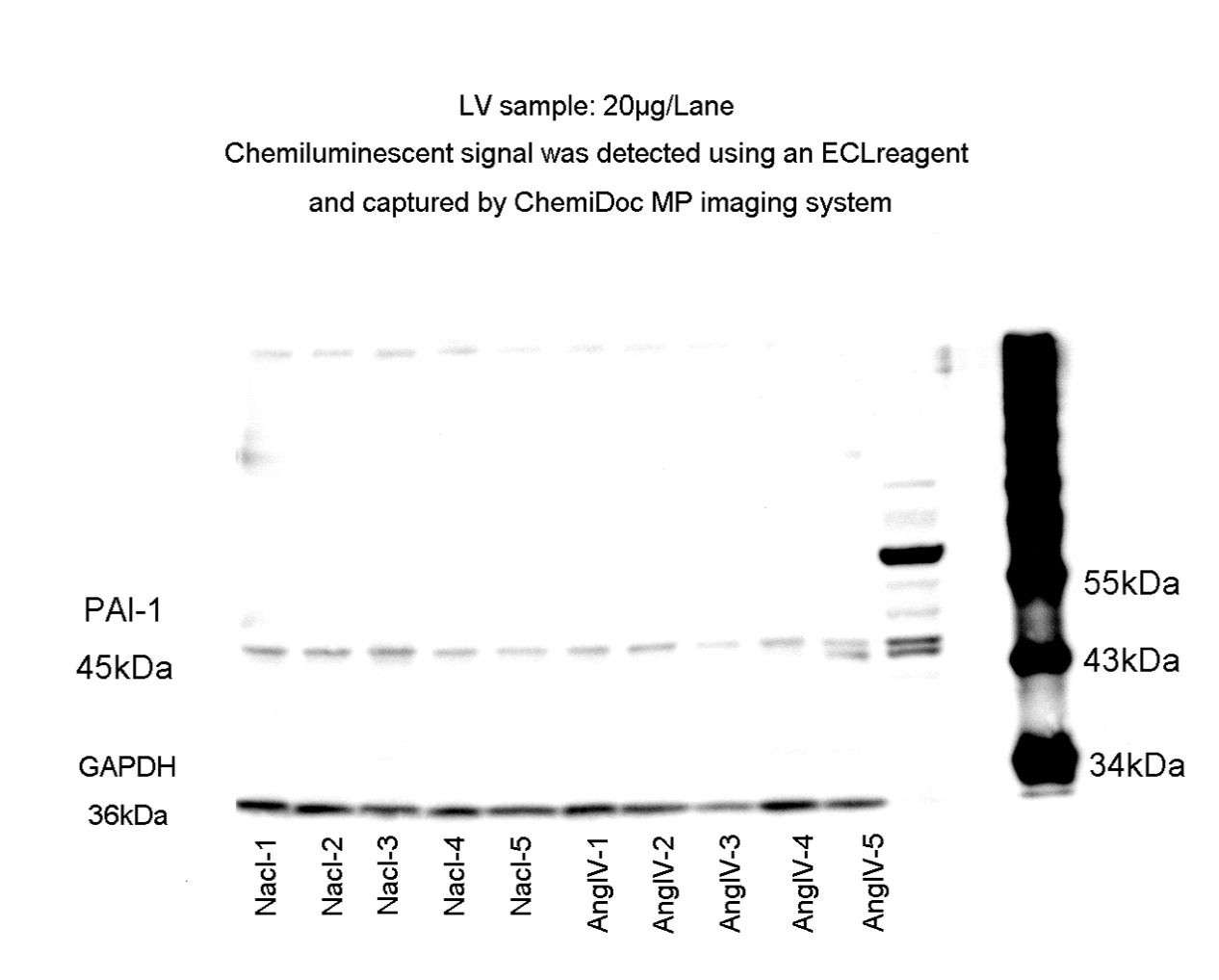
**

**Western blot results related to figure3E:**

**
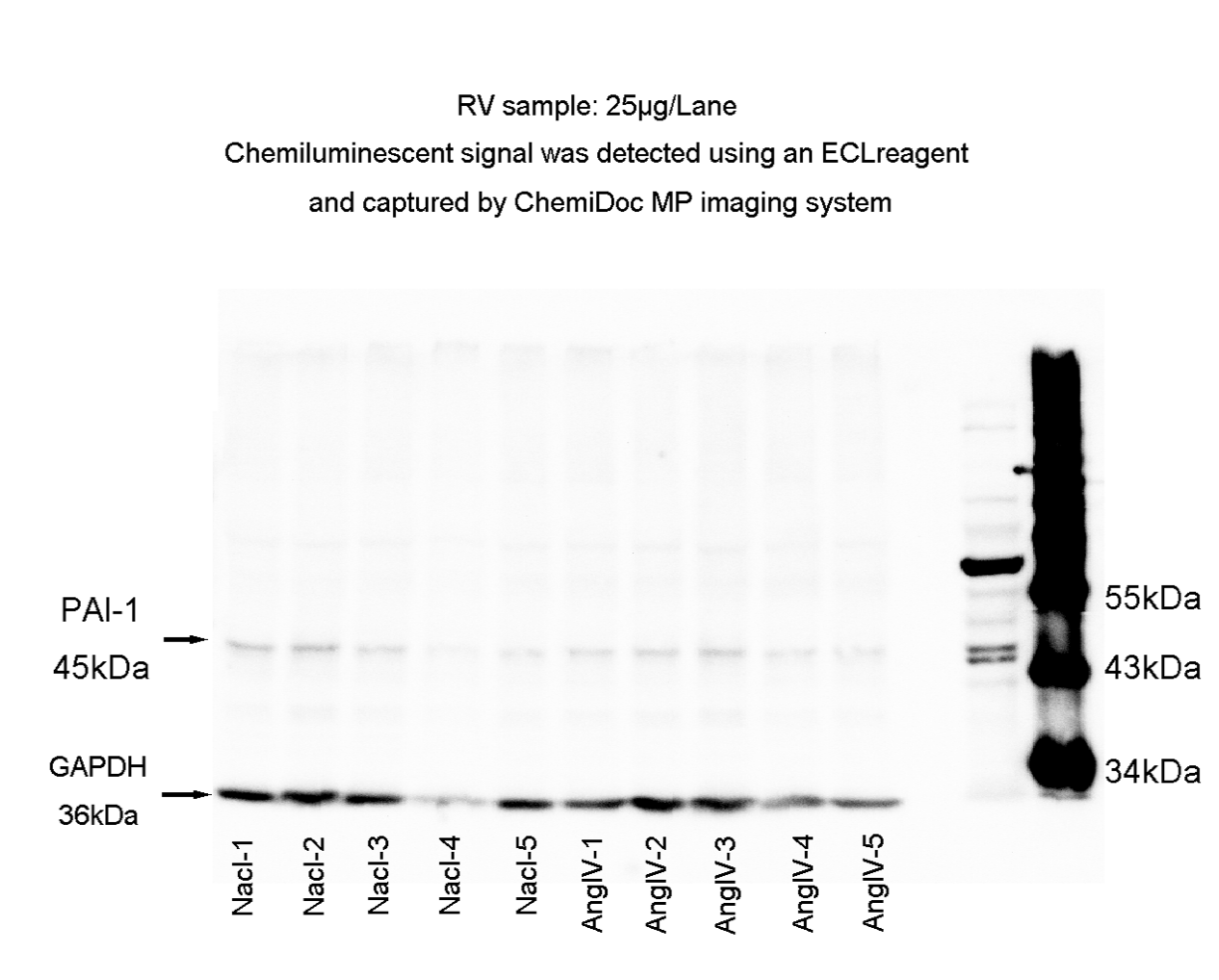
**

**Western blot results related to figure3F:**

**
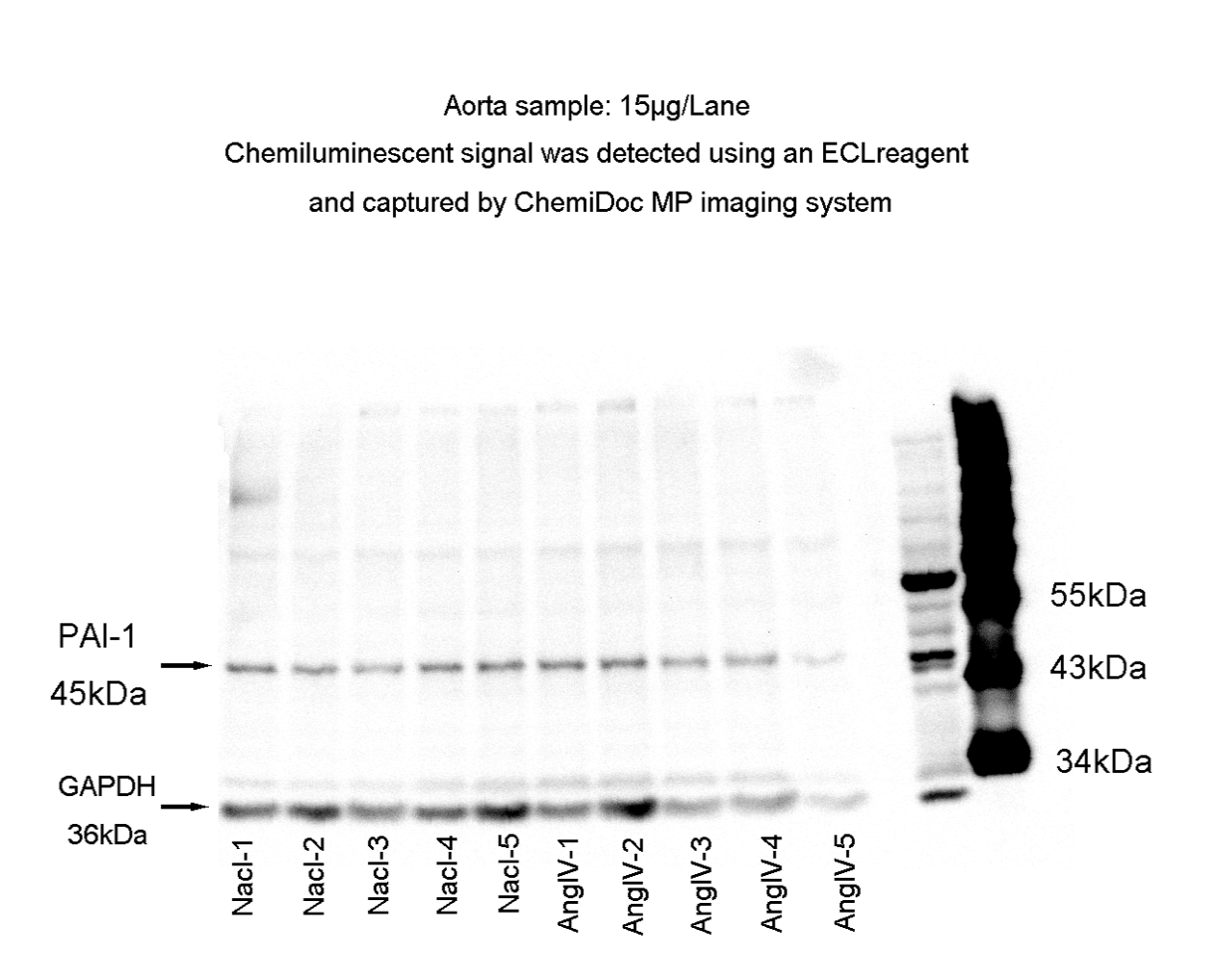
**
